# Olive stone as an eco-friendly bio-adsorbent for elimination of methylene blue dye from industrial wastewater

**DOI:** 10.1038/s41598-023-47319-x

**Published:** 2023-11-29

**Authors:** Saja M. Alardhi, Hussein G. Salih, Nisreen S. Ali, Ali H. Khalbas, Issam K. Salih, Noori M. Cata Saady, Sohrab Zendehboudi, Talib M. Albayati, Hamed N. Harharah

**Affiliations:** 1 Nanotechnology and Advanced Materials Research Center, University of Technology-Iraq, Baghdad, Iraq; 2 Department of Chemical Engineering, University of Technology-Iraq, 52 Alsinaa St., PO Box 35010, Baghdad, Iraq; 3 Materials Engineering Department, College of Engineering, Mustansiriyah University, Baghdad, Iraq; 4 Department of Chemical Engineering and Petroleum Industries, Al-Mustaqbal University, Babylon, 51001 Iraq; 5 Department of Civil Engineering, Memorial University, St. John’s, NL A1B 3X5 Canada; 6 Department of Process Engineering, Memorial University, St. John’s, NL A1B 3X5 Canada; 7 Department of Chemical Engineering, College of Engineering, King Khalid University, Abha, 61411 Kingdom of Saudi Arabia

**Keywords:** Chemical engineering, Environmental sciences

## Abstract

Adsorbents synthesized by activation and nanoparticle surface modifications are expensive and might pose health and ecological risks. Therefore, the interest in raw waste biomass materials as adsorbents is growing. In batch studies, an inexpensive and effective adsorbent is developed from raw olive stone (OS) to remove methylene blue (MB) from an aqueous solution. The OS adsorbent is characterized using scanning electron microscopy (SEM), Fourier Transform Infra-Red (FTIR), and Brunauer–Emmett–Teller (BET) surface area. Four isotherms are used to fit equilibrium adsorption data, and four kinetic models are used to simulate kinetic adsorption behavior. The obtained BET surface area is 0.9 m^2^ g^−1^, and the SEM analysis reveals significant pores in the OS sample that might facilitate the uptake of heavy compounds. The Langmuir and Temkin isotherm models best represent the adsorbtion of MB on the OS, with a maximum monolayer adsorption capacity of 44.5 mg g^−1^. The best dye color removal efficiency by the OS is 93.65% from an aqueous solution of 20 ppm at the OS doses of 0.2 g for 90 min contact time. The OS adsorbent serves in five successive adsorption cycles after a simple filtration-washing-drying process, maintaining MB removal efficiency of 91, 85, 80, and 78% in cycles 2, 3, 4, and 5, respectively. The pseudo second-order model is the best model to represent the adsorption process dynamics. Indeed, the pseudo second-order and the Elovich models are the most appropriate kinetic models, according to the correlation coefficient (R^2^) values (1.0 and 0.935, respectively) derived from the four kinetic models. The parameters of the surface adsorption are also predicted based on the mass transfer models of intra-particle diffusion and Bangham and Burt. According to the thermodynamic analysis, dye adsorption by the OS is endothermic and spontaneous. As a result, the OS material offers an efficient adsorbent for MB removal from wastewater that is less expensive, more ecologically friendly, and economically viable.

## Introduction

A growing worldwide interest has focused on developing wastewater treatment technologies to address the ever-increasing pollutants released into water, including industrial dyes^1–5^. The world uses about 7 million tonnes of dyes annually^6^. Moreover, dyes are used in the textile, food, paper, cosmetic, and pharmaceutical industries. During the dyeing process, 10 to 15% of these dyes reach the aquatic ecosystem^7^. Even though they may not enter aquatic habitats in most cases, their constant movement, because of the cumulative effect, poses a risk to aquatic ecosystems and the microorganisms that live there^8–10^. Existing conventional water treatment systems face a significant challenge due to the increasing rise in hazardous dye wastewater produced by various industrial/commercial sectors, which continues to be a serious public health problem and a top environmental protection priority^11^. Basic blue 9 is another name for methylene blue (MB), a cationic soluble dye or stain that can be found as a crystalline solid or a green/blue powder. MB is used in various fields, such as biology, medicine, and chemistry^12^. Wastewater is treated using different methods, such as membrane separation^13,14^, electrocoagulation^15,16^, flocculation^17^, precipitation^18^, ozonation^19^, and adsorption^20,21^. These well-established methods frequently fail to lower dye concentrations to desirable levels and are not economical for dye removal. Therefore, it is crucial to find treatment methods that are more efficient and economically feasible^12^. Adsorption is a technique capable of removing several types of pollutants from wastewater, including industrial dyes^22^. Nowadays, many adsorbents, including carbon-based nano-adsorbents^23,24^, transition metal-based oxides^25^, MOFs^26^, polymer-based adsorbents^27^, and low-cost adsorbents are used to treat dye-containing wastewater. It is possible to use inexpensive adsorbents from various sources, such as agricultural waste biomass, to remove contaminants from wastewater^28,29^. For wastewater treatment, agricultural wastes and cellulosic biomass hold tremendous promise as alternatives to activated carbon in adsorption^30,31^. Examples of low-cost adsorbents include pine leaves, pine cones, rice hull, tectona grandis sawdust, prickly pear seed cake, apricot seed, and sugarcane bagasse. These adsorbents showed promising results in removing several dyes such as Methylene Blue, Reactive Orange 16, Crystal Violet, Methyl Orange, Astrazone Black and Rhodamine B^21–27^. Table 1 presents the results of various reported studies of low-cost adsorbents on removal efficiency. Low-cost adsorbents demonstrated high removal efficiency (> 90%) for most of the dyes reported in Table 1.

**Table 1:** Removal efficiency of some dyes by different low-cost adsorbents.

Low-cost adsorbents	Dye	Removal efficiency (%)	Reference
Pine leaves	Methylene blue	~ 80	^21^
Pine cone	Methylene blue	63.8–94.8	^22^
Rice hull	Reactive orange 16	21.7–56.2	^23^
Tectona grandis sawdust	Crystal violet	~ 95	^24^
Prickly pear seed cake	Methyl orange	~ 98	^25^
Apricot seed	Astrazone black	62–91	^26^
Sugarcane bagasse	Rhodamine B	87.1–99.1	^27^

The olive stone is a lignocellulosic biomass with lipids, polysaccharides, phenols, hemicelluloses, and celluloses as the main constituents^32^. There are many innovative uses for olive stones. An fascinating and expanding utilization of olive stones is as heavy metal and dye adsorbents in wastewater treatment facilities^33^.

This work focuses on using olive stone as a low cost bio-waste material, and efficient adsorbent for removal of methylene blue (MB) dye from wastewater in a batch adsorption system under various experimental settings/conditions, due to its availability as a waste resource. The olive stones are produced from locally available material that can be reused for removing the environmental pollution by using the low price, and environmentally friendly bio-adsorbent material. The determination analytic methods of XRD, SEM, BET, and FTIR are utilized to analyze the adsorption data. In addition, Freundlich and Langmuir adsorption isotherms are examined for finding the most suitable isotherm model. The adsorption kinetics is also studied. Furthermore, the mass transfer and thermodynamic studies are investigated.

## Materials and methods

### Sample collection and preparation

Olive stones (OS) are collected from local markets in Baghdad. Initially, the OS sample is washed several times with distilled water to remove any impurities on the OS surface. Then, the OS sample is dried in an oven at 110 °C for 24 h. After drying, the OS sample is ground (pulverized) using a mechanical mill, sieved into a fine powder, and kept in a moisture-proof container for further assessment.

### Characterization of OS

The phases of crystalline materials are investigated using X-ray Diffraction (XRD) (type: Shimadzu-6000, origin: Japan) from 0° to 90° 2 theta degree with scan rate 2 (deg/min) and Cu–K (as a radiation source). FTIR spectroscopy (FTIR, Bruker Tensor 27, origin: Germany) is used to determine the functional groups on the surface of the adsorbent. The surface morphology for the OS is observed under a scanning electron microscope (SEM) (type: Tescan VEGA 3 SB, origin: Germany). The sample surface area is tested via BET instrument (Q-surf 9600, made in the USA).

### Preparation of MB stock solution

A 1 g of MB (C_16_H_18_ClN_3_S; MW = 319.85 g gmol^−1^) is dissolved in 1 L of distilled water to create a stock solution. Several initial MB concentrations (blank, 20, 40, 60, 80, 100, 120, and 140 mg L^−1^) are prepared. Under 663 nm, the MB calibration curve is performed by measuring the absorbance for each concentration (model JASCO V-630 Japan). From Eqs. (1) and (2), the dye removal from the solution and the adsorption capacity (*q*_*e*_) can be estimated^34:^

1 $$\mathrm{\% Removal}=\frac{{C}_{o}-{C}_{e}}{{C}_{o}}\times 100$$

2 $${q}_{e}=\frac{\left({C}_{o}-{C}_{e}\right)V}{M}$$

where *C*_*o*_ and *C*_*e*_ are the initial and equilibrium concentrations of adsorbate (mg L^−1^), respectively, $V$ denotes the volume (L) of the solution, $M$ is the mass (g) of the adsorbent, and ${q}_{e}$ introduces the amount of the contaminant adsorbed (mg g^−1^).

### Batch adsorption experiments

The batch adsorption experiments can evaluate several operational parameters, including the initial concentration, pH (2, 3, 5, 6, 7, 8, 9, and 12), temperature (25, 35, and 45 °C), and initial dye concentration (20–140 mg L^−1^). A 0.1 M of sodium hydroxide (NaOH) and/or hydrochloric acid (HCl) are used to adjust the pH. A shaker with a temperature control (Shaking Incubator, MODEL: SSI10R2) is utilized to shake 0.14 g of the OS adsorbent and 50 mL of MB solution at various starting concentrations for 24 h before centrifuging the mixture at 40,000 rpm. After each sample is vacuum-filtered through a 0.22 polycarbonate membrane, no dye tint is observed in the filter membrane. The MB concentration is measured using an ultraviolet–visible (UV) spectrophotometer.

### Isotherm and kinetic adsorption of methylene blue

Three isotherm and kinetic adsorption models are used to represent the adsorbent's capacity, as given in Table 2, and to better comprehend the kinetics of the adsorption behaviors^35^.

**Table 2:** Adsorption isotherm, kinetic, and mass transfer models.

Type	Models	Equations	Parameters	Ref
Isotherm	Langmuir	$\frac{{C}_{e}}{{q}_{e}}=\frac{1}{{q}_{max}}{C}_{e}+\frac{1}{{q}_{\mathit{max}} b}$	*C*_*e*_ (mg L^−1^) is the MB equilibrium concentration in the solution, and *q*_*e*_ (mg g^−1^) is the MB equilibrium amount eliminated. The maximum adsorption capacity is *q*_*max*_ (mg g^−1^), and the Langmuir constant is b (L (mg/g)^−1^)	^32^
	Freundlich	$\mathit{ln} {q}_{e} =\mathit{ln}{K}_{f}+\frac{1}{n}\mathit{ln}Ce$	*K*_*f*_ ^1/n ^(mg g^−1^)/(mg L^−1^) : MB's adsorption capacity, *n* is the factor of heterogeneity	^33^
	Dubinin–Radushkevich	$\mathit{ln} {q}_{e} =\mathit{ln}{q}_{D}-{B}_{D}.$ƹ_*D*_^2^	q_D_ is the maximum adsorption capacity in (mg g^−1^), *B*_*D*_ is the free energy coefficient of the adsorption (mol^2^/kJ^2^), ƹ_*D*_ is the Polanyi potential (kJ mol^−1^)	^34^
	Temkin	${q}_{e} = B ln {K}_{t} + B ln Ce$	*K*_*t*_ is the constant referring to the equilibrium (l g^−1^), *B* is the heat of adsorption (expressed in kJ mol^−1^), *R* is the universal gas constant (8.314 J mol^−1^ K^−1^), and *T* is the absolute temperature in Kelvin. *B* (J kJ^−1^) is a Temkin constant equal to (*RT/B*)	^35^
Kinetic	Pseudo-first-order	$\mathrm{log}\left({q}_{e}-{q}_{t}\right)=\mathrm{ log}{q}_{e}-\frac{{K}_{1}}{2.303} t$	*q*_*e*_ (mg g^−1^) is the equilibrium adsorption uptake, *q*_*t*_ (mg g^−1^) is the amount of the removed MB at time *t*, and *K*_1_ (min^−1^) is the rate constant of the first-order adsorption	^36^
	Pseudo-second-order	$\frac{t}{{q}_{t}}= \frac{1}{{K}_{2} {{q}_{e}}^{2}}+ \frac{1}{{q}_{e}} t$	Second-order adsorption rate constant, *K*_2_ (g (mg min)^−1^)	^37^
	Elovich model	${q}_{t}=A+B\mathrm{ln}t$	*A* is the adsorption constant, and *B* is the initial rate of adsorption (mg g^−1^ min^−1^)	^38^
Mass transfer	Intra-particle diffusion	${q}_{t}={K}_{P}{ t}^{0.5 }+I$	The constant of intra-particle diffusion rate is K_P_ (mg/(g min^0.5^)), and the constant of intra-particle diffusion is I	^43^
	Bangham and Burt model (BB)	$\mathrm{log log}\left[\frac{{C}_{i}}{{C}_{i}-{q}_{t} m}\right]=\mathrm{ log}\left[\frac{{K}_{b}m}{ 2.303V}\right]+ \alpha \mathrm{log}(t)$	*m* is the adsorbent mass, and *V* is the solution volume. Moreover, ${K}_{b}$ and α are the BB equation constants	^44^

### Mass transfer

Adsorption, a mass transfer process, is the phenomenon of gases or solutes sticking to solid or liquid surfaces. Due to unequal forces, the molecules or atoms on the solid surface adsorb because they have excess surface energy^36,37^. The adsorption of contaminants on the adsorbent surface occurs in three steps^40^: (1) contaminants move from the liquid film's boundary layer to the adsorbent surface as explained by the extra-particle diffusion model, (2) molecules diffuse either within the pores, below the surface, or both as explained by the intra-particle diffusion theory, and (3) contaminants adsorb through electrostatic interaction and hydrogen bonding in a surface chemical reaction. Because the third step is the fastest one, the first and second steps of the surface adsorption process can influence the adsorption amount and rate. It should be noted that all three phases, occurring separately or simultaneously, contribute to the adsorption process.

The Weber and Morris (WM) model, also known as the intra-particle diffusion model, uses the intra-particle diffusion coefficient to determine the mass transfer rate of contaminant particles to the interior of the synthetic adsorbent^38^. The mechanism of surface mass transfer to the adsorbent pores is investigated using the WM model. The theoretical fundamental is Fick's mass transfer law. In practice, the pore-based diffusion of the particle may control the adsorption process. Normally, the adsorption process can be regulated by the diffusion of the particles inside the pores. The Bangham and Burt (BB) model can be effective in these cases^39^. Table 2 tabulates the mass transfer models.

### Thermodynamics investigation

Another crucial aspect of adsorption studies is thermodynamics. The spontaneity and thermodynamic feasibility of the process are evaluated using thermodynamic parameters such entropy change (ΔSº), enthalpy change (ΔHº), and Gibbs free energy (ΔGº), as given below^40^:

3 $$\Delta {G}^{o}=-RTln{K}_{C}$$

where *K*_*c*_ represents the apparent equilibrium constant of the adsorption, expressed as follows^41^:

4 $${K}_{C}=\frac{{q}_{e}}{{C}_{e}}$$

The standard thermodynamic equilibrium constant (*K*_*c*_) of the adsorption system should be calculated using the activity rather than the concentration.

5 $$\Delta {\text{G}}^{{\circ}} \, = \,\Delta {\text{H}}^{{\circ}} {-}{\text{T }}\Delta {\text{S}}^{{\circ}}$$

Equation (6) describes the relation between the entropy and enthalpy, as given below^42^:

6 $$ln{K}_{C}=\frac{\Delta S^\circ }{R}-\frac{\Delta H^\circ }{RT}$$

## Results and discussion

### BET and scanning electron microscopy (SEM) analysis

**Figure 1:**
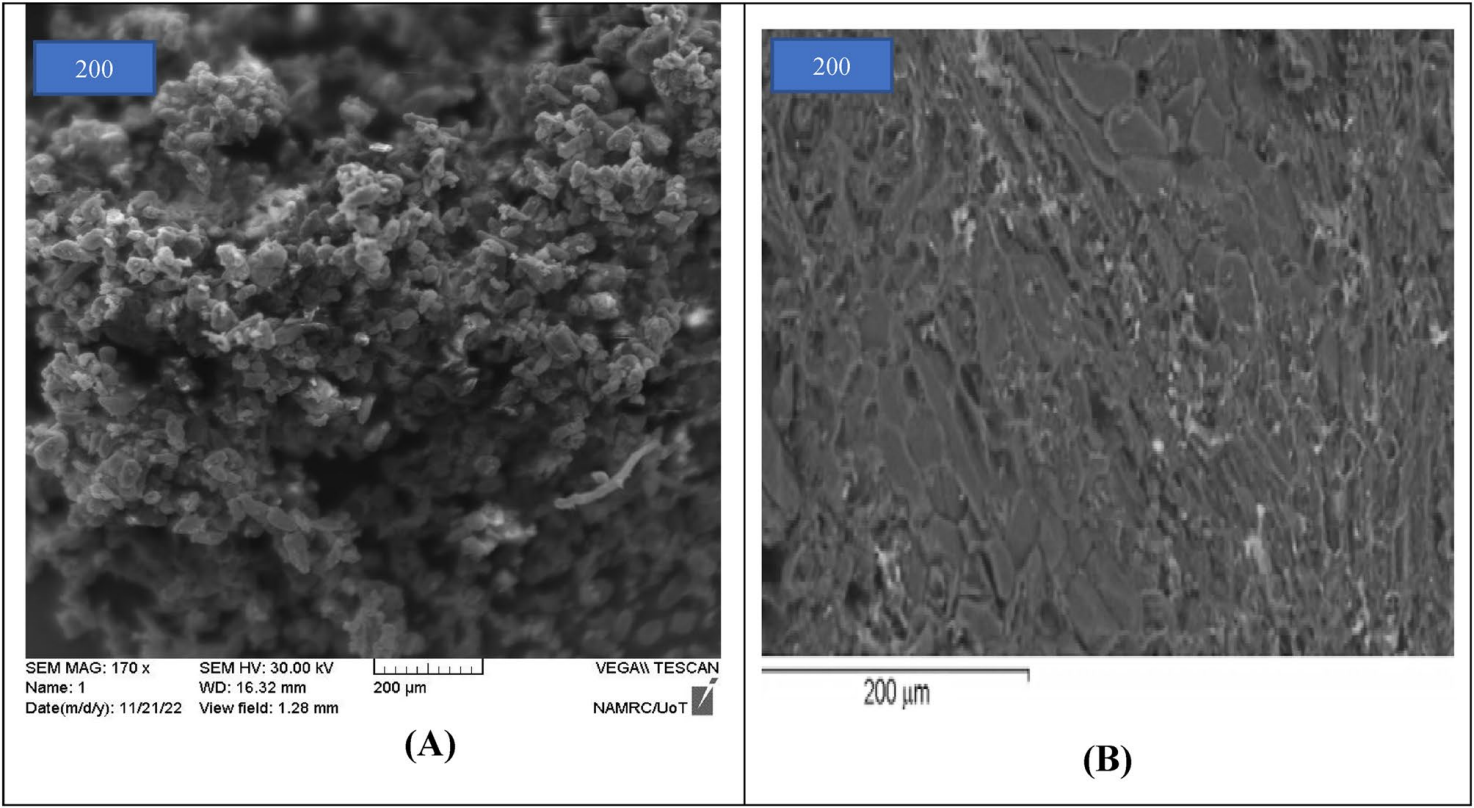
SEM image of olive stone: (**A**) before MB adsorption and (**B**) after MB adsorption.

The area of the olive stone (OS) sample is an irregular porous structure with average well-developed deep cavities and pores of diverse sizes and forms; the OS area is 0.9 m^2^ g^−1^. The SEM image of the OS powder is shown before MB adsorption in Fig. 1A, and after MB adsorption in Fig. 1B. The OS has a disorder structure, which favors the bio sorption of MB on different locations of the bio sorbent. This image confirms the previous results obtained by the analysis of the pore space/area of the OS samples^48^. Based on Fig. 1B, the particles of MB accumulation after adsorption can be seen, because of the adsorption of MB in the vacancies of the OS adsorbent material. It also demonstrates that the OS material is porous, giving MB molecules a considerable surface area to interact with and facilitate adsorption.

### X-ray diffraction (XRD)

**Figure 2:**
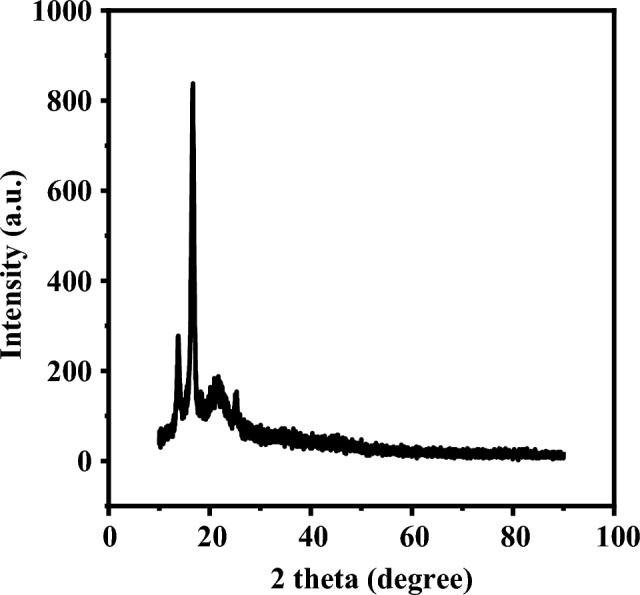
XRD patterns of olive stone adsorbent.

X-ray diffraction (XRD) is used to assess and validate the phase structures of the OS adsorbent. Figure 2 depicts the crystalline structure of OS powder, which is consistent with the findings of Tezcan et al.^43^, stating that the OS contains cellulose. Due to hydrogen bonding or van der Waals forces between nearby molecules, the final sample has a crystalline structure^44^. The similar results reveal the presence of a peak at 2θ = 19.85°, which is attributed to traces of lignin or hemicellulose^45^.

### Fourier-transform infrared spectroscopy

**Figure 3:**
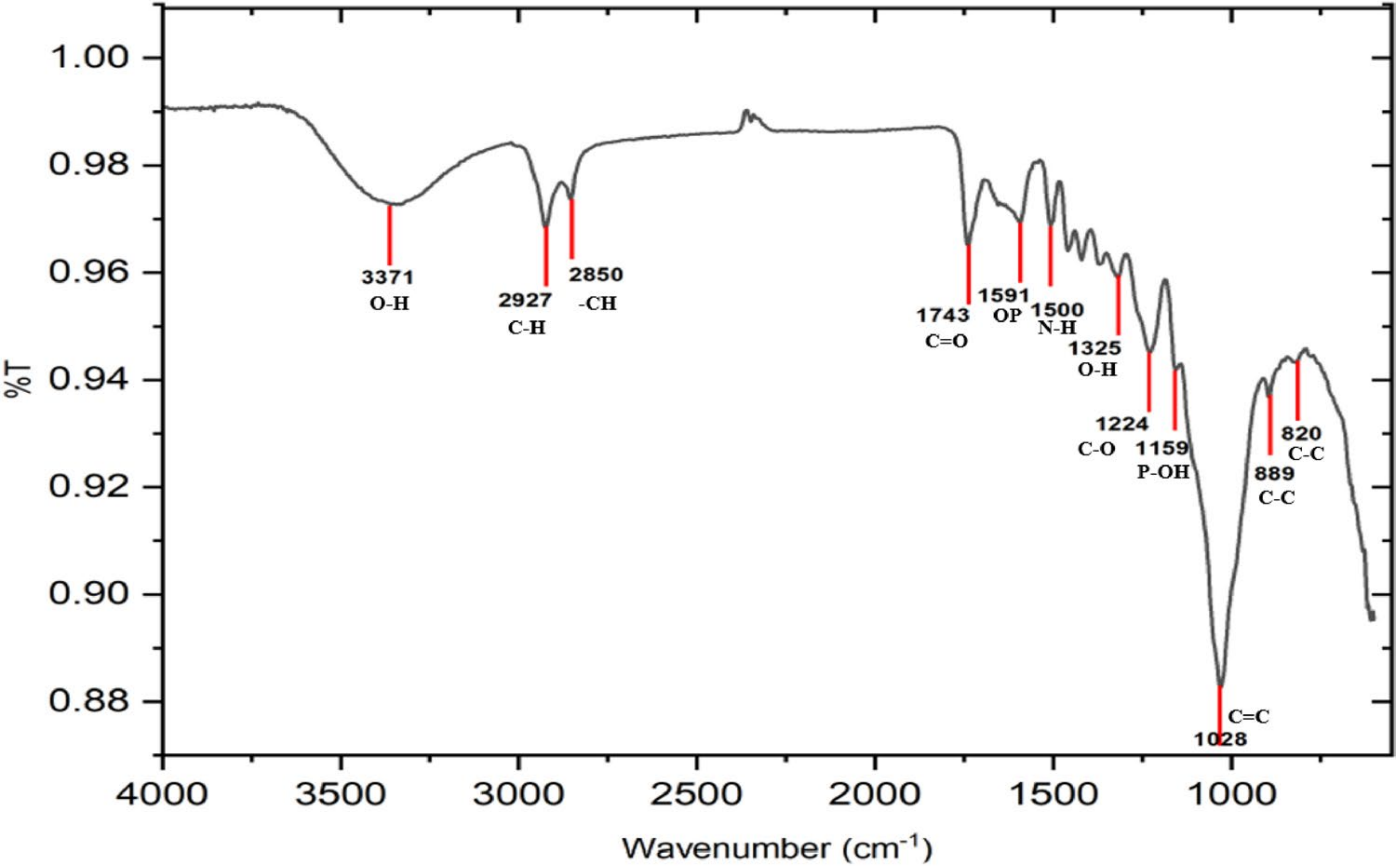
FTIR spectra of the olive stone adsorbent.

Figure 3 provides the FTIR spectrum for the OS sample. The first broad peak spanning at 3371 cm^−1^ is identified as the O–H hydroxyl with a single H bond. The band at 2927 cm^−1^ shows symmetric and asymmetric stretching of the C–H alkane. Furthermore, the existence of hemicellulose is suggested by the C=O carbonyl stretching at 1743 cm^−1^. The bands can be attributed to C–O stretching and C=C aromatic groups at 1224 cm^−1^ and 1028 cm^−1^, respectively^46^. The presence of carboxyl groups in the composite components causes the peaks at 1743 cm^−1^ noticed in the OP spectra^47^. P-OH stretching vibrations are detected by the absorption maxima at 1159 cm^−1^^48^. Figure 3 illustrates how the peaks at approximately 1591 cm^−1^ in the composites' spectra demonstrate the availability of OP within the composite^49^. The peaks at 2800–2900 cm^−1^ are noticeably amplified in accordance with symmetric vibrations of –CH. These peaks reveal the hydrogen bonds between the –CH and water or other hydroxyl groups in glycerol^50^. O–H stretching at 3371 cm^−1^ and O–H in plane bending at 1325 cm^−1^ are also observed, and N–H bending is noticed at 1500 cm^−1^^51^. Inorganic groups or C–C stretching vibrations are responsible for the other bands seen at 820 and 889 cm^−1^^52^.

### Effects of adsorption parameters

#### Effect of pH

**Figure 4:**
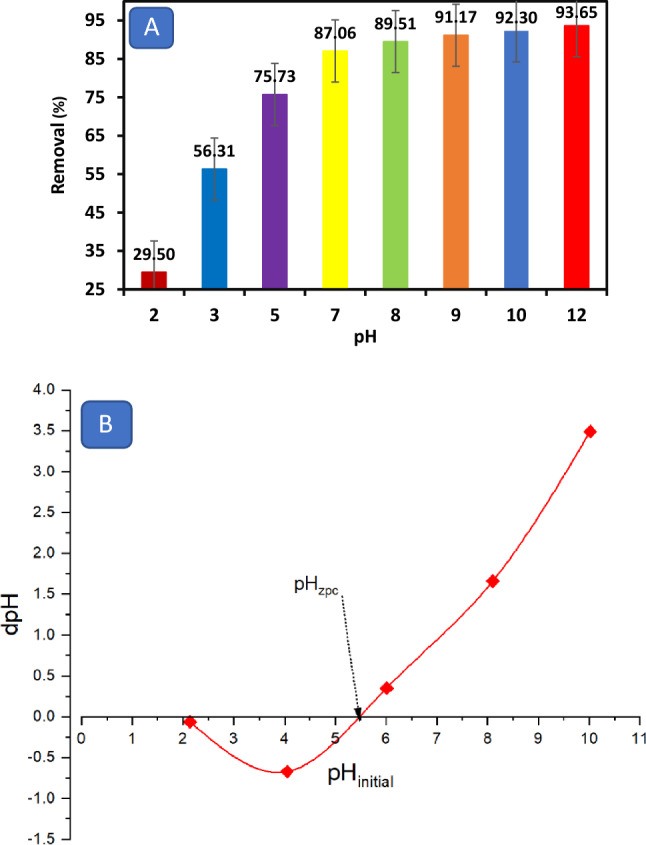
**(A)** Effect of pH on methylene blue adsorption using olive stones, and **(B)** point zero charge (pH pzc) for olive stone adsorbent.

It is obvious that MB adsorption is pH-dependent. Figure 4A demonstrates that when the pH is increased from 2 to 12, the MB adsorption on the OS increases from 29.5% to around 93%. This shows that the adsorption process favors an alkaline environment. The surface deprotonation might cause this phenomenon/behavior due to the presence of hydroxyl ions on the adsorbent's surface, which has a strong negative charge^46^. The weak attraction of the MB-positive ions to the protonated hydroxyl and/or carbonyl groups on the adsorbent surface may lead to the low removal efficiency. In other words, because hydrogen ions compete with the adsorbent for active sites, the electrostatic attraction between the cation dye and adsorbent surface is not strong enough^53^. According to Mulugeta and Lelisa^54^, more active sites for adsorption become available by the negative charges on the adsorbent surface. Numerous investigations have also revealed that this advantageous adsorption occurs around pH of 8–10^55–58^.

The point of zero charge (pHpzc) can be employed to evaluate the surface charge characteristics of the OS particles and the interaction between the functional groups of MB and the adsorbent. The isoelectric point (IEP) of the adsorbent is determined to be 5.5, at which point the net surface charge becomes neutral (Fig. 4B). When the pH falls below 5.5, the adsorbent acquires a positive charge due to the hydrogen bond formation, potentially impeding the adsorption of MB by causing repulsive forces. Conversely, when the pH increases above 5.5 (e.g., 9), the OS surface becomes negatively charged, promoting the adsorption of cationic MB through electrostatic interactions^59^. This favorable basic environment can be attributed to the presence of OH and COO^−^ groups on the adsorbent's surface.

#### Effect of adsorbent dosage

**Figure 5:**
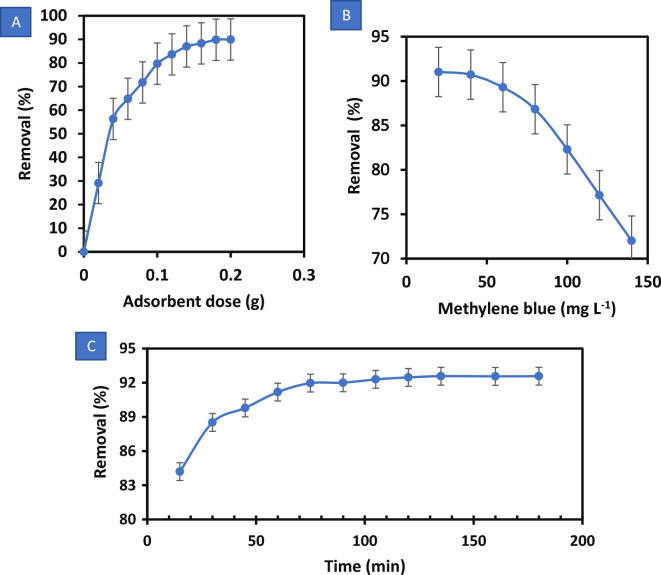
Methylene blue (MB) dye removal by olive stone adsorbent: (**A**) effect of olive stones dose, (**B**) effect of the MB concentration at 25 °C, and (**C**) effect of contact time.

For a given initial concentration of adsorbate, the amount of adsorbent plays a significant role in determining the sorption capacity^60^. Figure 5A depicts the adsorption of MB dye (20 mg L^−1^) as a function of OS dosage. Increasing the adsorbent dose increases the percentage of dye removal. It is clear that increasing the adsorbent dose increases the number of accessible adsorption sites, which increases the extent of adsorbed dye. Adsorption density decreases upon an increase in the adsorbent dose, because adsorption does not completely saturate adsorption sites^61^. Another possibility is that the high adsorbent dose causes inter-particle interactions, such as aggregation, which would reduce the adsorbent's overall surface area and lengthen the diffusional path^62^.

#### Impact of initial MB concentration

Figure 5B shows how the initial MB concentration affects the adsorption of MB onto OS. The highest MB removal percentage is found to be at 20 mg L^−1^ equal to 91.02% for the OS adsorbent. The adsorption of MB decreases somewhat, reaching 89.31% and 86.83% at 60 and 80 mg L^−1^ of the MB dye, respectively. At higher concentrations, the amount of dye removal drops accordingly. This is as a result of the adsorbent surface's unoccupied binding sites being available^63^. The removal effectiveness for the OS declines to 72.03% at 140 mg L^−1^, as seen in Fig. 5B. The inhibitory effects of the high dye concentration may be responsible for the low decolorization percentage and gradual drop in the MB removal at high concentrations^64^, due to a reduction in adsorption's required active sites and an increase in mass transfer's driving force^65^. Low MB dye concentrations can speed up and improve the adsorption process, but high initial concentrations hold the dyes molecules in the solution by solubilizing them. Additionally, it is found that adsorption equilibrium is reached more quickly and with a higher extent of MB adsorption at lower initial dye concentrations. This result could be explained by the lower initial concentration of MB molecules becoming spontaneously accessible to the open active sites on the surface of the OS adsorbent^66^.

#### Effect of contact time

Over time intervals ranging from 15 to 180 min, the impact of contact time on the adsorption of MB dye on OS adsorbent is examined. The adsorption extent as a function of contact time is shown in Fig. 5C; the removal efficiency of MB dye by the OS increases with increasing contact time, demonstrating how time affects the adsorption process^67^. According to Fig. 5C, it peaks at around 90 min and then is nearly stabilized for the remaining 180 min. Thus, it can be concluded that 90 min would be a sufficient time to achieve equilibrium in subsequent trials. Hence, this time period is adopted for all subsequent observations.

Studying the key process variables demonstrates that OS is an effective material in removing the MB dye, with a good removal efficiency equal to 93.65% compared to other low-cost adsorbents such as pine leaves^21^ and pine cone^22^ with the removal efficiencies equal to ~ 80% and 63.83–94.82%, respectively, (Table 1).

### Regeneration of adsorbent

**Figure 6:**
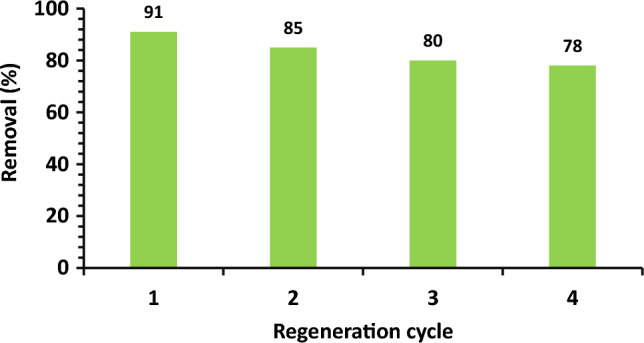
Regeneration of olive stone adsorbent for adsorption of MB.

During the regeneration phase, the OS used for adsorption is separated by filtration, washed with deionized water to remove residual MB, and finally dried at room temperature. Then, 200 mg of the spent adsorbent is added to 50 mL of 0.1 M HCL and stirred for 6 h. Later, the adsorbent is separated, washed, and oven-dried at 110 C for 3 h prior to being utilized in the regeneration cycles. The regeneration results are reported in Fig. 6. It is clear that the regeneration process of the adsorbent is successful, noting that the adsorption effectiveness decreases by a moderate level in the four cycles. Therefore, it can be concluded that the substance is effective even if it is used more than once.

### Isotherm and kinetic investigation

#### Isotherm and equilibrium models

**Figure 7:**
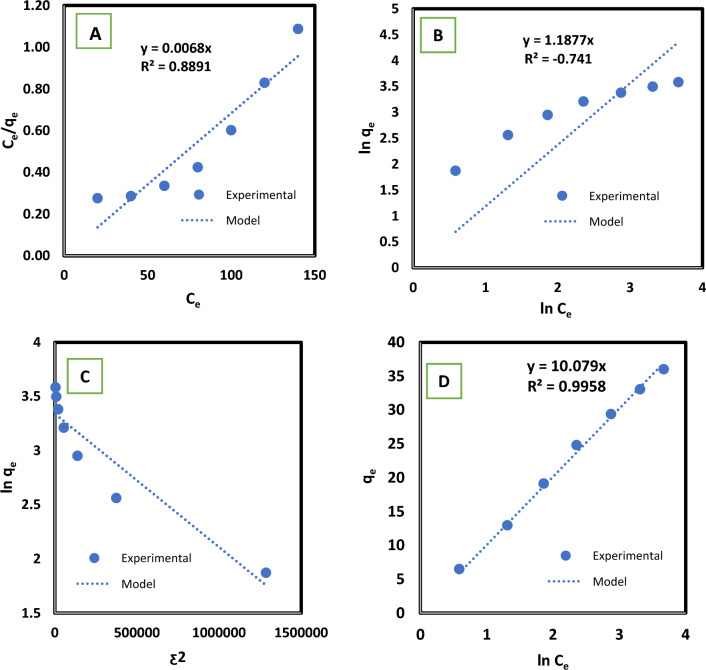
Isotherm models for methylene blue adsorption by olive stone: (**A**) Langmuir, (**B**) Freundlich, (**C**) Dubinin–Radushkevich, and (**D**) Temkin.

The adsorption isotherm model can explain the equilibrium uptake at a specific temperature for a certain adsorbent–adsorbate pair ^68^. The Langmuir, Freundlich, Dubinin–Radushkevich, and Temkin isotherms are examined in this study. The best-fit model is chosen based on the correlation coefficient (*R*^*2*^) value. Table 3 and Fig. 7 provide the results of various models considered in this study.

**Table 3:** Values of parameters for isotherm models.

Langmuir				Freundlich			Dubinin–Radushkevich		Temkin		
q_max_	R_L_	K_L_	R^2^	K_f_	1/n	R^2^	ƹ_*D*_	R^2^	B	K_t_	R^2^
44.5	0.316	0.108	0.996	5.929	0.534	0.921	0.637	0.883	9.77	1.09	0.997

Among the models that fit the equilibrium data, the best ones are Langmuir and Temkin models. The R_L_ value indicates the shape of the isotherm. Mckay et al.^69^ stated that RL values between 0 and 1 indicate favorable adsorption. In the present study, R_L_ value for the MB dye adsorption equals 0.316. Therefore, the adsorption process is favorable. The maximum monolayer coverage capacity *q*_*max*_ = 44.5 mg g^−1^ with a correlation coefficient *R*^2^ = 0.9968. Temkin isotherm assumes that all molecules' adsorption heat will drop linearly as the adsorbent surface is covered in more molecules due to the adsorbent-adsorbate interactions. This model assumes that, up to a particular maximum binding energy, the adsorption is described by a regular distribution of binding energies in the layer^70^.

#### Adsorption kinetics

**Figure 8:**
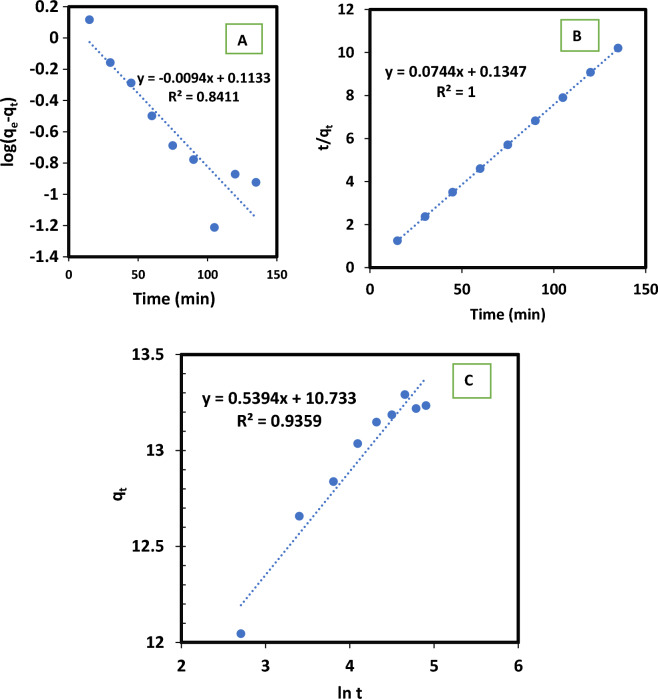
Kinetic models for methylene blue adsorption on olive stone: (**A**) pseudo first order, (**B**) pseudo second order, and (**C**) Elovich.

Table 4 and Fig. 8 provide the calculated parameters for various kinetics models as well as the validation results. Based on the outcomes, the Elovich and pseudo-second order models can accurately forecast the kinetics of MB adsorption onto the OS sample. The rate of solute adsorption is assumed proportional to the accessible sites on the adsorbent, according to the pseudo second-order model. The solute amount on the adsorbent surface determines the reaction rate, and the driving force is proportional to the total number of active sites present on the adsorbent surface^71^. Elovich model helps in predicting a system's activation and deactivation energy as well as mass and surface diffusion. The model assumes that as the amount of deposited solute increases, the rate of solute adsorption reduces exponentially^72^.

**Table 4:** Kinetics models for methylene blue adsorption on olive stone.

Pseudo-first order			Pseudo-second order			Elovich kinetic model		
q_e_ (mg g^−1^)	K_1_ (min^−1^)	R^2^	q_e_ (mg g^−1^)	K_2_ (g mg^−1^ min^−1^)	R^2^	*A*	*B*	R^2^
1.297	0.023	0.841	13.45	0.041	1	10.733	0.539	0.935

### Mass transfer models

This study presents the primary stage in the analysis of the mass transfer of surface adsorption process to forecast the properties of the OS adsorbent and the surface adsorption behavior. The two mass transfer models (WM and BB) for the surface adsorption of dye molecules on the adsorbent are shown in Table 5 which implies that the experimental results suite the model well. The model constant and R^2^ values are displayed in Table 5. These values indicate that a rate-controlling step is necessary for the diffusion of MB molecules into the pores of the adsorbent.

**Table 5:** Surface adsorption characteristics from two mass transfer models.

Intra-particle diffusion			Bangham and Burt		
I	K_p_ (mg/g min^0.5^)	R^2^	*K*_*b*_ (mL/g L)	*α*	R^2^
11.783	0.1418	0.8414	0.0138	0.0434	0.9311

### Adsorption thermodynamics

It is important to calculate thermodynamic properties such as enthalpy, entropy, and Gibbs free energy in an adsorption process. Thermodynamic calculations are conducted for the investigated adsorbent at 25, 35, and 45 °C with a fixed MB concentration. At 25, 35, and 45 °C, the ∆G° values for the OS adsorbent case are determined to be −16, −20, and −23 kJ mol^−1^, respectively. In addition, the values of ∆H° and ∆S° are 70 kJ mol^−1^ K and 290 J mol^−1^ K^−1^ , respectively. Based on the negative values found for Gibbs free energy, it can be concluded that the MB adsorption on the OS is spontaneous at all temperatures^73^. Positive entropy values further support the spontaneity and highly irregular behavior of the adsorption process. The endothermic nature of the MB adsorption process is explained by the positive values of the enthalpy while dealing with the OS sample^74^. This is also confirmed by the findings related to the impact of temperature on the adsorption of MB onto OS. Table 6 provides the thermodynamic parameters.

**Table 6:** Thermodynamic parameters for the adsorption of methylene blue onto olive stone.

Temp (°K)	Δ*H*° (kJ mol^−1^ K)	Δ*G*° (J mol^−1^)	Δ*S*° (J mol^−1^ K^−1^)
298	70	−16	290
308		−20	
318		−23	

### Mechanisms of adsorption

Four steps are often involved in the mechanism of MB reduction by adsorption on OS^69^:(1) bulk diffusion, or the movement of the MB component from the solution's bulk to the adsorbent surface; (2) MB dye molucules transfer to the adsorbent surface across the boundary layer (film diffusion); (3) transport of the MB dye molucules from the particle's surface inside its interior pores (also known as intra-particle or pore diffusion); and (4) MB adsorption through physical adsorption using van der Waals forces at an active location on the material's surface^70^. To better understand the MB adsorption mechanisms, the kinetic and isotherms results are obtained. It makes the following assumptions in light of the Langmuir correlation coefficient (R^2^ = 0.996): (1) adsorption occurs in a monolayer; (2) a finite (fixed) number of equal and equivalent definite localized sites cannot host adsorption; and (3) even on nearby sites, steric hindrance and lateral contact between the adsorbed molecules can develop. The n value determines the Freundlich isotherm adsorption intensity; n = 2–10, n = 1–2, and n < 1 correspond to good features, moderate adsorption, and bad adsorption, respectively^71^. Consequently, the intra-particle diffusion and heterogeneous surface are confirmed by the Freundlich isotherm with 0.4 < n < 1, which is in line with the Temkin isotherm. The adsorption of the MB chemical by OS occurs in two phases, as shown by the kinetics of the intra-particle diffusion model and the impact of contact time: (1) the adsorption of MB compounds occurs on the OS external surface, which is the instantaneous adsorption stage (film diffusion); and (2) the movement of MB compound within the OS pores is confined (particle diffusion). According to Table 5, in addition to the surface adsorption characteristics, mass transfer mechanisms, such as intra-particle diffusion and external diffusion, and adsorption thermodynamics can contribute to the interactions between the MB compound and OS particles. The creation of an intra-bond between the OS and MB chemical controls the adsorption process on the OS in addition to surface interaction, particularly with low specific area. This explains the high adsorption capacity achieved based on the Temkin isotherm. The adsorption mechanisms are generally complicated and may include additional elements including temperature, ion exchange interactions, and dispersive force^71,72^.

## Conclusions

Olive stone, a waste product, is an inexpensive adsorbent capable of removing methylene blue (MB) dye from aqueous solutions. This study assesses the removal of MB dye through batch tests. The MB dye adsorption on the OS follows the pseudo-second-order and the Elovich models based on the results and statistical analysis of various models. Modeling the adsorption isotherms with Langmuir and Temkin models offers a better fit. Additionally, the mass transfer mechanism of the surface adsorption for dye molecules on the OS adsorbent is examined using two mass transfer models. In the experimental phase of MB dye wastewater treatment, the tests are conducted to evaluate the effects of the solution pH, adsorbent dose, dye initial concentration, adsorption time, and temperature on the adsorption amount. According to the thermodynamic analysis, dye adsorption by the OS is endothermic and spontaneous. Overall, olive stone (as an adsorbent) effectively removes MB dye from aqueous solutions. Thus, it is a good choice for this purpose in wastewater treatment. This is because chemically-processed adsorbents entail pollution risks. As a result, OS offers an adsorbent for MB removal from wastewater that is low-cost, more ecologically friendly, and economically viable. Systematic tests might be implemented successfully to increase the use of OS and sustainably treat MB-contaminated wastewater at small to large scales. For future work, it is recommended to conduct the adsorption tests at broad ranges of temperatures, pressures, concentrations/compositions, and types of contaminants in various water and wastewater streams.

## Data Availability

All relevant data and material are presented in the main paper.

## References

[1] Ali NS, Harharah HN, Salih IK, Cata Saady NM, Zendehboudi S, Albayati TM (2023). Applying MCM-48 mesoporous material, equilibrium, isotherm, and mechanism for the effective adsorption of 4-nitroaniline from wastewater. Sci. Rep..

[2] Humadi JI, Jafar SA, Ali NS (2023). Recovery of fuel from real waste oily sludge via a new eco-friendly surfactant material used in a digital baffle batch extraction unit. Sci. Rep..

[3] Ali NS, Salih IK, Harharah HN, Majdi HSh, Salih HG, Kalash KR, Al-Shathr A, Al-Sudani FT, Abdulrahman MA, Alrubaye JM (2023). Utilization of loaded cobalt onto MCM-48 mesoporous catalyst as a heterogeneous reaction in a fixed bed membrane reactor to produce isomerization product from *n*-heptane. Catalysts.

[4] Mahdi AE, Ali NS, Majdi HSh, Albayatia TM, Abdulrahman MA, Jasim DJ, Kalash KR, Salih IK (2023). Effective adsorption of 2-nitroaniline from wastewater applying mesoporous material MCM-48: Equilibrium, isotherm, and mechanism investigation. Desalin. Water Treat..

[5] Khudhur RH, Ali NS, Khader EH, Abbood NS, Salih IK, Albayati TM (2023). Adsorption of anionic azo dye from aqeous wastewater using zeolite NaX as an efficient adsorbent. Desalin. Water Treat..

[6] Bhagat C, Kumar M, Tyagi VK, Mohapatra PK (2020). Proclivities for prevalence and treatment of antibiotics in the ambient water: A review. npj Clean Water.

[7] Ali SS, Sun J, Koutra E, El-Zawawy N, Elsamahy T, El-Shetehy M (2021). Construction of a novel cold-adapted oleaginous yeast consortium valued for textile azo dye wastewater processing and biorefinery. Fuel.

[8] Maged A, Iqbal J, Kharbish S, Ismael IS, Bhatnagar A (2020). Tuning tetracycline removal from aqueous solution onto activated 2:1 layered clay mineral: Characterization, sorption and mechanistic studies. J. Hazard. Mater..

[9] Ali NS, Majdi HSh, Albayati TM, Jasim DJ (2021). Adsorption of aniline from aqueous solutions onto a nanoporous material adsorbent: Isotherms, kinetics, and mass transfer mechanisms. Water Pract. Technol..

[10] Mahdi AE, Ali NS, Kalash KR, Salih IK, Abdulrahman MA, Albayati TM (2023). Investigation of equilibrium, isotherm, and mechanism for the efficient removal of 3-nitroaniline dye from wastewater using mesoporous material MCM-48. Prog. Color Colorants Coat..

[11] Ali NS, Kalash KR, Ahmed AN, Albayati TM (2022). Performance of a solar photocatalysis reactor as pretreatment for wastewater via UV, UV/TiO_2_, and UV/H_2_O_2_ to control membrane fouling. Sci. Rep..

[12] Jabbar NM, Alardhi SM, Mohammed AK, Salih IK, Albayati TM (2022). Challenges in the implementation of bioremediation processes in petroleum-contaminated soils: A review. Environ. Nanotechnol. Monit. Manag..

[13] Khadim AT, Albayati TM, Cata Saady NM (2022). Removal of sulfur compounds from real diesel fuel employing the encapsulated mesoporous material adsorbent Co/MCM-41 in a fixed-bed column. Microporous Mesoporous Mater..

[14] Hamedi H, Ehteshami M, Mirbagheri SA, Rasouli SA, Zendehboudi S (2019). Current status and future prospects of membrane bioreactors (MBRs) and fouling phenomena: A systematic review. Can. J. Chem. Eng..

[15] Ali NS, Alismaeel ZT, Majdi HSh, Salih HG, Abdulrahman MA, Cata Saady NM, Albayati TM (2022). Modification of SBA-15 mesoporous silica as an active heterogeneous catalyst for the hydroisomerization and hydrocracking of *n*-heptane. Heliyon.

[16] Al-Jaaf HJ, Ali NS, Alardhi SM, Albayati TM (2022). Implementing eggplant peels as an efficient bio-adsorbent for treatment of oily domestic wastewater. Desalin. Water Treat..

[17] Abbood NS, Ali NS, Khader EH, Majdi HSh, Albayati TM, Cata Saady NM (2023). Photocatalytic degradation of cefotaxime pharmaceutical compounds onto a modified nanocatalyst. Res. Chem. Intermediat..

[18] Mohammed MY, Ali AM, Albayati TM (2022). Choline chloride-based deep eutectic solvents for ultrasonic-assisted oxidative desulfurization of actual heavy crude oil. Chem. Eng. Res. Des..

[19] Abd Al-Khodor YA, Albayati TM (2023). Real heavy crude oil desulfurization onto nanoporous activated carbon implementing batch adsorption process: Equilibrium, kinetics, and thermodynamic studies. Chem. Afr..

[20] Muslim WA, Albayati TM, Al Nasri SK (2022). Decontamination of actual radioactive wastewater containing 137Cs using bentonite as a natural adsorbent: Equilibrium, kinetics, and thermodynamic studies. Sci. Rep..

[21] Hamedi H, Zendehboudi S, Rezaei N, Azizi A, Shahhoseini F (2023). Application of functionalized Fe(3)O(4) magnetic nanoparticles using CTAB and SDS for oil separation from oil-in-water nanoemulsions. Langmuir.

[22] Gutiérrez M, Grillini V, Mutavdžić Pavlović D, Verlicchi P (2021). Activated carbon coupled with advanced biological wastewater treatment: A review of the enhancement in micropollutant removal. Sci. Total Environ..

[23] Bezerra de Araujo CM, Filipe Oliveira do Nascimento G, Rodrigues Bezerrada Costa G, Santos da Silva K, Salgueiro Baptisttella AM, Gomes Ghislandi M, Alves da Motta Sobrinho M (2019). Adsorptive removal of dye from real textile wastewater using graphene oxide produced via modifications of hummers method. Chem. Eng. Commun..

[24] Al-Jadir T, Alardhi SM, Al-Sheikh F, Jaber AA, Kadhim WA, Rahim MHA (2023). Modeling of lead (II) ion adsorption on multiwall carbon nanotubes using artificial neural network and Monte Carlo technique. Chem. Eng. Commun..

[25] Dubey K, Dubey S, Sahu V, Modi A, Bamne J, Haque FZ, Gaur NK (2023). Defects and oxygen vacancies modified properties of transition metal doped Ce0.95X0.05O_2_ (X = Fe Co, Ni) nanoparticles. Mater. Sci. Eng. B.

[26] Uddin MJ, Ampiaw RE, Lee W (2021). Adsorptive removal of dyes from wastewater using a metal-organic framework: A review. Chemosphere.

[27] Moradi O, Sharma G (2021). Emerging novel polymeric adsorbents for removing dyes from wastewater: A comprehensive review and comparison with other adsorbents. Environ. Res..

[28] Karić N, Maia AS, Teodorović A, Atanasova N, Langergraber G, Crini G, Ribeiro ARL, Đolić M (2022). Bio-waste valorisation: Agricultural wastes as biosorbents for removal of (in)organic pollutants in wastewater treatment. Chem. Eng. J. Adv..

[29] dos Santos KJL, de Souza dos Santos GE, de Sá ÍMGL, de Carvalho SHV, Soletti JI, Meili L, da Silva Duarte JL, Bispo MD, Dotto GL (2019). Syagrus oleracea-activated carbon prepared by vacuum pyrolysis for methylene blue adsorption. Environ. Sci. Pollut. Res..

[30] Pang X, Sellaoui L, Franco D, Dotto GL, Georgin J, Bajahzar A, Belmabrouk H, Ben Lamine A, Bonilla-Petriciolet A, Li Z (2019). Adsorption of crystal violet on biomasses from pecan nutshell, para chestnut husk, araucaria bark and palm cactus: Experimental study and theoretical modeling via monolayer and double layer statistical physics models. Chem. Eng. J..

[31] dos Santos KJL, dos Santos GEDS, de Sá ÍMGL, Ide AH, Duarte JLDS, de Carvalho SHV, Soletti JI, Meili L (2019). Wodyetia bifurcata biochar for methylene blue removal from aqueous matrix. Bioresour. Technol..

[32] Espadas-Aldana G, Vialle C, Belaud J-P, Vaca-Garcia C, Sablayrolles C (2019). Analysis and trends for Life Cycle Assessment of olive oil production. Sustain. Prod. Consumpt..

[33] Boubakri S, Djebbi MA, Bouaziz Z, Namour P, Jaffrezic-Renault N, Amara ABH, Trabelsi-ayadi M, Ghorbel-Abid I, Kalfat R (2018). Removal of two anionic reactive textile dyes by adsorption into MgAl-layered double hydroxide in aqueous solutions. Environ. Sci. Pollut. Res..

[34] Alardhi SM, Fiyadh SS, Salman AD, Adelikhah M (2023). Prediction of methyl orange dye (MO) adsorption using activated carbon with an artificial neural network optimization modeling. Heliyon.

[35] Mahdia AH, Jaida GM, Alardhib SM (2021). Artificial neural network modelling for the removal of lead from wastewater by using adsorption process. Desalin. Water Treat..

[36] Hu, H. & Xu, K. Chapter 8—Physicochemical technologies for HRPs and risk control. In *High-Risk**Pollutants**in**Wastewater* (Ren, H., Zhang, X. Eds.). 169–207. (Elsevier, 2020). 10.1016/B978-0-12-816448-8.00008-3.

[37] Nouri N, Tasviri M, Zendehboudi S (2023). Effect of poly(vinyl alcohol) on catalytic performance of Al-pillared clay in alkylation of aromatic hydrocarbons with olefins. Ind. Eng. Chem. Res..

[38] Cabooter D, Song H, Makey D, Sadriaj D, Dittmann M, Stoll D, Desmet G (2021). Measurement and modelling of the intra-particle diffusion and b-term in reversed-phase liquid chromatography. J. Chromatogr. A.

[39] Prajapati AK, Mondal MK (2020). Comprehensive kinetic and mass transfer modeling for methylene blue dye adsorption onto CuO nanoparticles loaded on nanoporous activated carbon prepared from waste coconut shell. J. Mol. Liq..

[40] De Castro MLFA, Abad MLB, Sumalinog DAG, Abarca RRM, Paoprasert P, de Luna MDG (2018). Adsorption of methylene blue dye and Cu(II) ions on EDTA-modified bentonite: Isotherm, kinetic and thermodynamic studies. Sustain. Environ. Res..

[41] Jua LY, Karri RR, Mubarak NM, Yon LS, Bing CH, Khalid M, Jagadish P, Abdullah EC (2020). Modeling of methylene blue adsorption using functionalized buckypaper/polyvinyl alcohol membrane via ant colony optimization. Environ. Pollut..

[42] Awad AM, Jalab R, Benamor A, Nasser MS, Ba-Abbad MM, El-Naas M, Mohammad AW (2020). Adsorption of organic pollutants by nanomaterial-based adsorbents: An overview. J. Mol. Liq..

[43] Sounni F, Aissam H, Ghomari O, Merzouki M, Benlemlih M (2018). Electrocoagulation of olive mill wastewaters to enhance biogas production. Biotechnol. Lett..

[44] Zhang YH, Lynd LR (2004). Toward an aggregated understanding of enzymatic hydrolysis of cellulose: Noncomplexed cellulase systems. Biotechnol. Bioeng..

[45] Allaoui S, Bennani MN, Ziyat H, Qabaqous O, Tijani N, Ittobane N, Hodaifa G (2021). Valorization of crude olive stone in the removing of polyphenols from crude olive mill wastewater: Kinetic, isotherm and mechanism study. Heliyon.

[46] Malekbala MR, Hosseini S, Kazemi Yazdi S, Masoudi Soltani S, Malekbala MR (2012). The study of the potential capability of sugar beet pulp on the removal efficiency of two cationic dyes. Chem. Eng. Res. Des..

[47] Akar T, Tosun I, Kaynak Z, Ozkara E, Yeni O, Sahin EN, Akar ST (2009). An attractive agro-industrial by-product in environmental cleanup: Dye biosorption potential of untreated olive pomace. J. Hazard. Mater..

[48] Han MH, Yun Y-S (2007). Mechanistic understanding and performance enhancement of biosorption of reactive dyestuffs by the waste biomass generated from amino acid fermentation process. Biochem. Eng. J..

[49] Kaya N, Atagur M, Akyuz O, Seki Y, Sarikanat M, Sutcu M, Seydibeyoglu MO, Sever K (2018). Fabrication and characterization of olive pomace filled PP composites. Compos. Part B Eng..

[50] Jabeen B, Rafique U (2014). Synthesis and application of metal doped silica particles for adsorptive desulphurization of fuels. Environ. Eng. Res..

[51] Gülel, Ş. & Güvenilir, Y. Using olive stone powder for biodegradation of bio-based polyamide 5.6. *Proceedings* **69**(1), 2 (2021).

[52] Amar MB, Walha K, Salvadó V (2020). Evaluation of olive stones for Cd(II), Cu(II), Pb(II) and Cr(VI) biosorption from aqueous solution: Equilibrium and kinetics. Int. J. Environ. Res..

[53] Manna S, Roy D, Saha P, Gopakumar DA, Thomas S (2017). Rapid methylene blue adsorption using modified lignocellulosic materials. Process Saf. Environ. Protect..

[54] Mulugeta M, Lelisa B (2014). Removal of methylene blue (Mb) dye from aqueous solution by bioadsorption onto untreated parthenium hystrophorous weed. Mod. Chem. Appl..

[55] Ovando-Medina VM, Díaz-Flores PE, Martínez-Gutiérrez H, Moreno-Ruiz LA, Antonio-Carmona ID, Hernández-Ordoñez M (2014). Composite of cellulosic agricultural waste coated with semiconducting polypyrrole as potential dye remover. Polymer Compos..

[56] Hassaan MA, Yılmaz M, Helal M, El-Nemr MA, Ragab S, El Nemr A (2023). Isotherm and kinetic investigations of sawdust-based biochar modified by ammonia to remove methylene blue from water. Sci. Rep..

[57] Egbosiuba TC, Abdulkareem AS, Kovo AS, Afolabi EA, Tijani JO, Auta M, Roos WD (2020). Ultrasonic enhanced adsorption of methylene blue onto the optimized surface area of activated carbon: Adsorption isotherm, kinetics and thermodynamics. Chem. Eng. Res. Des..

[58] Babaei AA, Alavi SN, Akbarifar M, Ahmadi K, Ramazanpour Esfahani A, Kakavandi B (2016). Experimental and modeling study on adsorption of cationic methylene blue dye onto mesoporous biochars prepared from agrowaste. Desalin. Water Treat..

[59] Ahmad R, Ansari K (2021). Comparative study for adsorption of congo red and methylene blue dye on chitosan modified hybrid nanocomposite. Process. Biochem..

[60] Hojati S, Landi A (2015). Removal of zinc from a metal plating wastewater using an Iranian sepiolite: Determination of optimum conditions. Desalin. Water Treat..

[61] Yu LJ, Shukla SS, Dorris KL, Shukla A, Margrave JL (2003). Adsorption of chromium from aqueous solutions by maple sawdust. J. Hazard. Mater..

[62] Shukla A, Zhang Y-H, Dubey P, Margrave JL, Shukla SS (2002). The role of sawdust in the removal of unwanted materials from water. J. Hazard. Mater..

[63] Vijayaraghavan K, Yun Y-S (2008). Biosorption of C.I. reactive black 5 from aqueous solution using acid-treated biomass of brown seaweed *Laminaria* sp. Dyes Pigments.

[64] Anjaneya O, Souche SY, Santoshkumar M, Karegoudar TB (2011). Decolorization of sulfonated azo dye Metanil Yellow by newly isolated bacterial strains: *Bacillus* sp. strain AK1 and *Lysinibacillus* sp. strain AK2. J. Hazard Mater..

[65] Pathania D, Sharma S, Singh P (2017). Removal of methylene blue by adsorption onto activated carbon developed from *Ficus*
*carica* bast. Arab. J. Chem..

[66] Marrakchi F, Auta M, Khanday WA, Hameed BH (2017). High-surface-area and nitrogen-rich mesoporous carbon material from fishery waste for effective adsorption of methylene blue. Powder Technol..

[67] Potgieter JH, Potgieter-Vermaak SS, Kalibantonga PD (2006). Heavy metals removal from solution by palygorskite clay. Miner. Eng..

[68] Muttakin M, Mitra S, Thu K, Ito K, Saha BB (2018). Theoretical framework to evaluate minimum desorption temperature for IUPAC classified adsorption isotherms. Int. J. Heat Mass Transf..

[69] Azaman SH, Afandi A, Hameed B, Din AM (2018). Removal of Malachite green from aqueous phase using coconut shell activated carbon: Adsorption. Desorpt. Reusabil. Stud. J. Appl. Sci. Eng..

[70] Iqbal J, Wattoo FH, Wattoo MHS, Malik R, Tirmizi SA, Imran M, Ghangro AB (2011). Adsorption of acid yellow dye on flakes of chitosan prepared from fishery wastes. Arab. J. Chem..

[71] Tan KL, Hameed BH (2017). Insight into the adsorption kinetics models for the removal of contaminants from aqueous solutions. J. Taiwan Inst. Chem. Eng..

[72] George William, K., Serkan, E., Atakan, Ö., Özcan, H.K. & Serdar, A. Modelling of adsorption kinetic processes—Errors, theory and application. In *Advanced**Sorption**Process**Applications,**IntechOpen,**Rijeka*. Chap. 10 (Serpil, E. Ed.) (2018). 10.5772/intechopen.80495.

[73] Ebelegi AN, Ayawei N, Wankasi D (2020). Interpretation of adsorption thermodynamics and kinetics. Open J. Phys. Chem..

[74] Hasanpour M, Hatami M (2020). Application of three dimensional porous aerogels as adsorbent for removal of heavy metal ions from water/wastewater: A review study. Adv. Colloid Interface Sci..

